# 4167 Peri-transplant Lung Microbiome Reveal Oral Bacteria, Pepsin And Inflammatory Markers Co-associate With Primary Graft Dysfunction, Implicating Aspiration As A Potential Contributor

**DOI:** 10.1017/cts.2020.339

**Published:** 2020-07-29

**Authors:** John Evan McGinniss, Joshua M. Diamond, Melanie C. Brown, Ed Cantu, Jason D. Christie, Rick D. Bushman, Ronald G. Collman

**Affiliations:** 1 University of Pennsylvania School of Medicine

## Abstract

OBJECTIVES/GOALS: Primary graft dysfunction (PGD) is acute lung injury in the first three days after lung transplant. Patients that experience PGD have increased mortality and an increased risk of chronic lung allograft dysfunction. The pathogenesis is thought to be an ischemia-reperfusion injury but is incompletely understood and there are no specific therapies. We investigated the role of the microbiome in PGD and associations with inflammation and markers of aspiration. METHODS/STUDY POPULATION: We collected airway lavage samples from lung transplant donors before procurement and recipients after reperfusion. We extracted DNA, amplified the bacterial 16S rRNA gene, and sequenced on the Illumina MiSeq platform. QIIME2 and Deblur were used for bioinformatic analysis. R packages were used for downstream analysis and visualizations. The host response was quantified using the Milipore 41-plex Luminex and an ELISA for pepsin. Clinical data was collected by the Penn Lung Transplant Outcomes Group. PGD was assessed by degree of hypoxemia and chest X-ray findings in the 72 hours after transplant. RESULTS/ANTICIPATED RESULTS: There was no significant difference in alpha diversity (Shannon index, p = 0.51), biomass (via comparison of 16S amplicon PicoGreen, p = 0.6), or beta diversity (Weighted UniFrac, p = 0.472, PERMANOVA) between subjects with PGD grade 3 (n = 36) and those that did not (n = 96). On taxonomic analysis, we found an enrichment of Prevotella in donor and recipient lungs that went on to develop PGD (p = 0.05). To follow up this finding we measured immune response and pepsin concentrations in recipient lungs. We found elevated levels in 35/41 cytokines measured in subjects that developed PGD as well as an elevation in pepsin and a correlation between pepsin concentration and Prevotella relative abundance (Figure 1). Additionally, Prevotella relative abundance had statistically significant positive correlations with multiple cytokines such as IL-6 (Pearson’s = 0.26, p = 0.009) and eotaxin (Pearson’s = 0.24, p = 0.016). DISCUSSION/SIGNIFICANCE OF IMPACT: There is an enrichment of oral anerobes in lung allografts that eventually develop PGD. This is associated with elevated levels of pepsin and markers of inflammation. These lines of evidence suggest aspiration contributes to priming the allograft for PGD.

